# The influence of internship training experience on Kenyan and Ugandan doctors’ career intentions and decisions: a qualitative study

**DOI:** 10.1080/16549716.2023.2272390

**Published:** 2023-11-09

**Authors:** Yingxi Zhao, Daniel Mbuthia, Dos Santos Ankomisyani, Claire Blacklock, David Gathara, Sassy Molyneux, Catia Nicodemo, Tom Richard Okello, Elizeus Rutebemberwa, Raymond Tweheyo, Mike English

**Affiliations:** a NDM Centre for Global Health Research, Nuffield Department of Medicine, University of Oxford, Oxford, UK; b KEMRI-Wellcome Trust Research Programme, Nairobi, Kenya; c Uganda Virus Research Institute - International AIDS Vaccine Initiative HIV Vaccine Program (UVRI-IAVI), Entebbe, Uganda; d MARCH Centre, London School of Hygiene and Tropical Medicine, London, UK; e KEMRI-Wellcome Trust Research Programme, Kilifi, Kenya; f Nuffield Department of Primary Care Health Sciences, University of Oxford, Oxford, UK; g Department of Economics, Verona University, Verona, Italy; h Department of Surgery, Faculty of Medicine, Lira University, Lira, Uganda; i Department of Health Policy, Planning and Management, Makerere University School of Public Health, Kampala, Uganda; j Centre for Health Systems Research and Development (CHSRD), The University of Free State, Bloemfontein, South Africa

**Keywords:** Internship, medical education, career preference, workforce, labour market

## Abstract

**Background:**

Medical internship is a key period for doctors’ individual career planning and also a transition period for the broader labour market.

**Objectives:**

We aimed to understand the complex set of factors influencing the career intentions and decisions of junior doctors, post-internship in Kenya and Uganda.

**Methods:**

We conducted semi-structured interviews with 54 junior medical officers and 14 consultants to understand doctors’ internship experiences and subsequent employment experiences. We analysed the data using a mix of a direct content approach, informed by an internship experience and career intentions framework developed primarily from high-income country literature, alongside a more inductive thematic analysis.

**Results:**

Echoing the internship experience and career intentions framework, we found that clinical exposure during internship, work–life balance, aspects of workplace culture such as relationships with consultants and other team members, and concerns over future job security and professional development all influenced Kenyan and Ugandan doctors’ career preferences. Additionally, we added a new category to the framework to reflect our finding that interns might want to ‘fill a health system gap’ when they choose their future careers, based on what they witness as interns. However, often career intentions did not match career and employment decisions due to specific contextual factors, most importantly a shortage of job opportunities.

**Conclusion:**

We have shown how internship experiences shape medical doctors’ career intentions in Kenya and Uganda and highlighted the importance of job availability and context in influencing doctors’ career choices.

## Background

Medical internship is an essential element of post-graduate training, aiming to provide new doctors with a period of supervised work as they solidify their clinical competencies and develop confidence [1]. From an individual’s point of view, the internship years are a time when trainees also make their initial career decisions [2]. The short rotations between different clinical teams during internship can shape junior doctors’ perceptions of professional roles and identities and are important in influencing which speciality areas they enjoy most/feel most suited to, and where they might prefer to work, e.g. urban, rural, public, or private settings [3–5].

Ideally, countries accommodate these post-internship doctors within their health service, in careers addressing national and regional health system needs. For those trained in the public sector, ongoing employment within the national health system is desirable. As such, international migration could be seen as a system failure, but is now common in both high-income and low- and middle-income countries (LMICs). Indeed, almost two-thirds of interns trained in Ireland intended to migrate abroad to practice medicine [6]. Likewise, 19% of the UK foundation year (FY) 2 trainees planned to work outside the UK, take a career break or leave medicine permanently in 2019, pre-Covid [7]. For Kenya and Uganda, the absorption of post-internship medical officers into the public sector was as low as 45.8% [8] and 48.2% [9], respectively. Low absorption can reflect supply or demand-side challenges, as doctors may not want to work in the public sector, or there may be inadequate job opportunities [10]. In countries where speciality choices are made during or immediately after internship, challenges may also arise when there is an imbalance between supply and demand of posts in different specialities [11,12]. Exits from the public health service after internship are concerning because they represent loss of a significant financial investment [13], as medical education is typically highly subsidised by the public sector, especially in LMICs [14]. Indeed, migration to high-income countries immediately after qualification risks substantial financial losses in training costs [15–17]. These losses could be as high as US$ 127,221 per doctor, a total of US$ 2.2 billion for nine sub-Saharan African countries until 2010 according to Mills et al. [14].

In our previous meta-ethnographic review [10], we revealed a range of ways in which internship experience shapes medical doctors’ career intentions and decisions (Table 1) and produced a conceptual framework of this phenomenon. The internship experience and career intentions framework highlighted the importance of ensuring sufficient positive and inspiring clinical exposure, improving the workplace environment and culture, building supportive relationships with consultants and other senior team members and valuing all specialities. Additionally, it was found that changes in contractual, job market and training policies should be communicated clearly and early in the career path to provide more career security. However, most of the 23 included studies were from high-income countries, especially the UK, with only three from LMICs (India, Sierra Leone and South Africa), where internship experience was noted to be particularly stressful and where doctors may be more likely to migrate or leave the public sector [15,18].

**Table 1.** Framework from the meta-ethnography [10].

Theme	Subtheme/Category
Finding which career option is “more me”	1. The ‘hands-on’ experience and ‘real life’ exposure
	2. Positive experience, confidence and readiness
	3 .The workload, work–life balance, lifestyle
	4. Well-being, emotional stress and the need to step off
	5. Image of professions and self-identity
Exploring, experiencing and witnessing workplace and organisational norms	6. Relationship with supervisors/consultants
	7. Relationship with peers
	8. Relationship with senior colleagues and the team
	9. Relationship with patients and the community
	10. Characteristics and hierarchy of career options and specialties
	11. Workplace location, conditions, resources and environment
	12. Feeling valued by the organisation and healthcare system
Worrying about the future	13. Job market policies and changes, job security: will I get a job
	14. Future training and professional development opportunities: will I get advanced

Given the lack of empirical evidence to help better understand how internship experience may influence career intentions and decisions in LMICs, we wanted to explore the relevance of the internship experience and career intentions framework in two East African countries, Kenya and Uganda. In both countries, medical doctors/medical officers (MOs) are trained through a Bachelor of Medicine & Bachelor of Surgery (MBChB) programme that lasts 6 years, with an additional internship year. In Kenya the internship year is split into rotations in surgery, obstetrics and gynaecology [OBGYN], internal medicine and paediatrics, with psychiatry and community health added as two compulsory rotations in 2020. In Uganda, the new 5–1 policy requires interns to choose two ‘major’ specialities for 5-month rotations and two ‘minor’ specialities for 1-month rotations [19]. After completing the internship and subsequent licensure by regulators, individuals can practise medicine unsupervised as general medical doctors in public, private and private not-for-profit clinics and hospitals. Both countries have expanded medical school intakes to help meet national health workforce gaps over the past decades, but currently both have a 45–50% non-absorption rate of medical graduates post-internship into the public sector [8,9]. We analysed qualitative interview data in these two countries to better understand what is influencing career intentions and decisions of junior doctors in these settings and in so doing examined the relevance of our earlier developed framework.

## Methods

### Data collection

This study is part of broader work examining the internship and employment experiences of medical doctors in Kenya and Uganda. We conducted semi-structured interviews with both MOs and their supervising consultants. The latter were also included to provide us with perspectives on interns’ competence and how this may have changed. The semi-structured interview guide is provided in Supplementary appendix 1, and includes questions on internship experiences around wellbeing, educational and work environment, career and employment intentions and their influences. The guide is broad in-nature and was adapted iteratively throughout the study. The internship experience and career intentions framework did not inform the interview guide as the framework was developed after the interviews were completed.

We sampled the interviewees over June 2021 and June 2022 using a mix of convenience and snowball approaches. In Kenya we first identified participants through our personal networks and then asked them to inform other eligible participants across the country about the opportunity to participate in the study. In Uganda we recruited participants during health facility visits. We purposively sampled a mix of MOs who had completed their internship within the last 3 years, and who now worked in a range of different occupations and settings (in public, private or faith-based hospitals as MOs, working in research, business, or unemployed) as well as a mix of consultants from different specialities. Data collection was conducted by DM (a Kenyan male researcher) and YZ (a UK-based, Chinese male researcher) in Kenya, and by RT (a Ugandan male doctor and researcher) and 10 other trained short-term research assistants who were doctors or researchers in Uganda. All research assistants had experience of qualitative research. Interviews were conducted in English, either online or face-to-face at the health facilities, and lasted between 20 and 60 min. Participants were not previously known to the interviewers, and interviewers’ affiliations with Kenyan/Ugandan research institutions were disclosed to participants at the start of the interviews. No participants withdrew from the study. Data saturation was considered to be achieved during the data collection phase; however, some new themes were subsequently identified during analysis which might have been strengthened through additional data collection (such as probing questions around professional identities).

### Data analysis

Interviews were audio recorded and transcribed, with all personal identifiers removed and replaced by unique anonymous codes. Data were imported into NVivo (version 1.4.1) for analysis. Transcripts were not returned to participants for review. Since one of the main objectives of the study was to understand the usefulness of, and refine, the conceptual framework developed in our previous review [10] in the Kenyan and Ugandan setting, we compared and contrasted this framework with the exploratory interview data. We used a directed content approach to analyse the interview data, focusing on exploring career intentions [20]. Due to the timing of data collection activities, the Kenyan transcripts were analysed first. YZ first read through and coded the transcripts deductively based on the 15 categories developed in the framework. Where new themes emerged from the analysis that did not fit into the original framework, these were used to revise the framework through inductive coding and iterative discussions with other team members. A new category, i.e. ‘filling health systems gaps’, was added to the final framework. We also highlighted the similarities and differences under each category between the review framework and the Kenyan and Ugandan interview results (see supplementary appendix 2).

The other objective of the paper was to better understand how the specific contexts in Kenya and Uganda shape MOs’ career intentions vs. decisions [10]. The data were analysed and coded inductively using a thematic approach [21], with major themes refined and summarised as we familiarised ourselves with the data. Data analysis was led by YZ with refining of findings and interpretation though discussion with co-authors.

**Box 1.** Labelling of qualitative quotes.

We used the following labelling approach to indicate the sources of qualitative quotes in this paper. (1) For medical officer respondents, we labelled them as ‘respondent No. – Level of internship facility + facility ownership – Current job’. For example, ‘Kenyan medical officer, KM01 – L5 Public – Research’ refers to Kenyan medical officer 01 who interned in a level 5 public hospital and currently working in research. (2) For consultant respondents, we labelled them as ‘respondent No. – Level of current facility + facility ownership – Specialty’. For example, ‘Uganda consultant, UC01 – National Public – OBGYN’ refers to Uganda consultant 01 who works as an obstetrician and gynaecologist consultant in a public national hospital.

### Ethical approval

Ethical approval for the study was received from the Oxford Tropical Research Ethics Committee (OxTREC 563–20 and OxTREC 518–21), Kenya Medical Research Institute (KEMRI) (SERU 4071) and Makerere University School of Public Health, Research Ethics Committee and the Uganda National Council for Science and Technology in Uganda (SPH-2021-168 and HS2062ES). Written consent as well as verbal informed consent were obtained from interview participants.

### Reflexivity

This study is a collaboration between Kenyan, Ugandan and international researchers. The first authors (YZ and DM) are not doctors but have research experience in public health. The senior authors are a Ugandan male doctor and researcher (RT) and a UK male doctor and researcher with extensive experience working in Kenya (ME). YZ, DM, RT and ME co-designed the study including interview development and data interpretation. Discussions were held between YZ and DM during the early stages of data collection to guide later interviews and analysis. Together with the 10 Ugandan research assistants who were either doctors or researchers, and other Uganda-based and UK-based researchers, the research team included both ‘insiders’ and ‘outsiders’, facilitating rich discussions [22]. The emerging findings were sense-checked with peer audiences including Kenya and UK-based health systems researchers external to the project [23]. Seven of the authors (YZ, DM, CB, DG, SM, CN, ME) were involved in the original framework development that informed our coding and analysis, and this might have led to biases, which were mitigated by involving other researchers in the data analysis and sense-checking activities.

## Results

A total of 68 interviews were conducted with 30 Kenyan and 24 Ugandan MOs, as well as 10 Kenyan and 4 Ugandan consultants. Most of the Kenyan MOs at the time of the interviews were working in medicine, with others working in research, business or as specialist trainees. For Ugandan medical officers, nearly one-third of the interviewees was unemployed. In Kenya we included consultants from all four major specialities (surgery, internal medicine, paediatrics and obstetrics/gynaecology), while in Uganda we included consultants from obstetrics/gynaecology, surgery and paediatrics (Table 2).

**Table 2.** Semi-structured interview sample characteristics.

Medical officers	Kenya (*n *= 30)	Uganda (*n *= 24)
*Internship hospital type*	25 public, 3 private not for profit, 1 private, 1 military	14 public, 8 private not for profit, 2 private
*Internship hospital level*	14 level 5, 16 level 4	3 national referral, 11 regional referral, 10 general
*Current occupation*	26 MO, 2 researcher, 1 speciality resident, 1 in business	14 MO, 2 researcher, 1 speciality resident, 7 current unemployed
Consultants	Kenya (*n*=10)	Uganda (*n*=4)
*Internship hospital type*	8 public, 1 private not for profit, 1 private	3 public, 1 private not for profit
*Internship hospital level*	3 level 6, 4 level 5, 3 level 4	3 national, 1 general
*Speciality*	2 surgery, 2 internal medicine, 5 paediatric, 1 obstetrics/gynaecology	2 obstetrics/gynaecology, 1 surgery, 1 paediatric

Interviews with MOs showed that career intentions evolved over time. Some MOs had identified speciality choices as early as medical school, largely informed by their interactions with undergraduate lecturers and clerkship experiences. In Kenya this sometimes influenced their choice of internship centres – e.g. they would choose facilities with better surgical capacity. In Uganda due to the new 5–1 policy where interns have to choose two ‘major’ specialities (5 months) and two other ‘minor’ specialities (1 month), their early career intentions also influenced these choices. The following section describes the results of the direct content analysis.

### Theme 1: finding which career option is ‘more me’

As found in the previous framework, ‘hands-on experience’, ‘real-life exposure’ or continuing medical education (CME) sessions (framework category 1) were found to influence MOs’ career intentions during the internship.

Because internship puts you in the actual workplace. When you’re in medical school, you are seeing things from outside almost … So when you go into internship, you’re like in the real life medical situation, you have decisions to make, you’re the one in charge of particular tasks and stuff like that. So it makes you see how you could potentially be like living for the rest of your life. (Ugandan medical officer, UMO21 – General public – Private MO)

The categories of ‘Positive experience, confidence and readiness’ (original category 2) in the previous framework were also supported by the data; good experiences in certain rotations fostered interest (e.g. experiences during COVID-19 led to more interests in infectious diseases), whereas negative experiences (e.g. due to Covid-19-related lockdowns and facility closure) turned interns’ interest away from specific specialities. In Kenya, when interns perceived they had insufficient exposure or experience in certain areas, they sometimes tried to fill these perceived gaps in their training by applying for specific post-internship MO jobs. In contrast, a lack of confidence due to perceived inadequate experience during internship would discourage some Ugandan MOs from further advancing in those specialities.

I had wanted to major in surgery because I feel like … I really [had] not exhausted my potential during my internship at the surgery department, so I wanted to start with surgery so that I can learn as much as I can but that was not possible then. (Kenyan medical officer, KMO02 – L5 Public – Public MO)

Actually right now I can’t do anything in surgery, I am not confident enough. It is more of biasing for example you go for something you are not passionate about for five months and then get just a month for something you are passionate about. I was in the surgical ward but with no theatre during Covid-19. we just did medical management of surgical patients. (Uganda medical officer, UMO12 – Regional public – Unemployed)

‘Workload, work-life balance, lifestyle’ (original category 3) and ‘well-being, emotional stress and the need to step off’ (original category 4) were found to be relevant to the Kenyan and to a lesser extent Ugandan MOs. Most MOs intended to work in careers with more manageable workloads, based on their observation of their senior colleagues during internship. Some specialities were observed to be very stressful, with seniors ‘living on the edge’ which made these specialities less desirable to MOs. The difference in workload between public, private and faith-based facilities was consistently mentioned by Kenyan and Ugandan MOs but was not captured in the original framework. For example, respondents reported that workloads as interns were highest in the public sector, however for post-internship MOs in the public sector the workloads were perceived to be comparatively lower.

Because of the way internship is set up, you get burnt out a lot and then you just become [normal]- when you start applying for jobs, you become really sceptical about working too many hours because you don’t want to ever be that burnt out again. (Ugandan medical officer, UMO17 – General private not for profit - unemployed)

Data from Kenya and Uganda supported ‘professional image and self-identity’ (original category 5) as relevant. Indeed, for Kenyan and Ugandan trainees, some specialities were considered ‘not for doctors’ but rather for clinical officers (non-physician clinicians) or nurses. However, this finding was not strongly supported by data.

… guys don’t like doing anaesthesia. They feel that there is no work for them, they only work in ICUs, you know there was a course that was introduced, it is for clinical officers. So they specialise in anaesthesia and then nursing, nurses can also do, become an anaesthesia nurses. (Kenyan medical officer, KMO20 – L4 Public – Private not for profit MO)

### Theme 2: Exploring, experiencing, and witnessing workplace and organisational norms

Kenyan and Ugandan MOs also reported that relationships with colleagues influenced their career intentions. ‘Relationship with consultants’ (original category 6) was consistently emphasised as the most important factor for many MOs. Positive influence was linked to receiving encouragement, mentorship and career advice from consultants and thus ‘wanting to be like them’. Conversely, negative influence was associated with witnessing bad role modelling, such as consultants displaying bullying behaviours or lacking professionalism. These influences were identified in the original framework. MOs also reported that as interns they witnessed and observed consultants who they considered to be either successful or struggling which impacted their intentions.

But then when I transfer transition to internal medicine, it was it was a hell… I was racially profiled and at some point, it almost generated to physical abuse … So what end up happening, in as much as I loved internal medicine, I knew I deep down wanted to be a physician … I left [Hospital N] knowing I will never, ever, ever touch internal medicine again, because I didn’t want to be liked to those two bullies. (Kenyan medical officer, KMO08 – L4 Private – Private MO)

While the Kenyan and Ugandan data also suggested that interns talked to peers to understand and share working experiences in different hospitals and potential job opportunities (‘relationship with peers’, original category 7), we did not identify any data specifically supporting the concepts of ‘validation’ by peers, maintaining ‘personal relationships’ or competition with peers identified in the previous framework. As for ‘relationship with senior colleagues and the team’ (original category 8), similar to the review findings, interns drew on the experiences and advice of former interns. Some MOs were also inspired by nurses in their careers. Registrars (senior MOs) were not identified as influential on the career intentions of interns in the Kenyan data but were found to be influential in the Uganda data and the previous framework. This may be due to registrars only working in a small number of tertiary hospitals in Kenya. Findings on ‘relationship with the patient/community’ (original category 9) were similar between the original framework and the interview data. For example, Ugandan data suggested that some interns did not enjoy patient interaction, and therefore preferred a career that would not involve direct patient care.

I was lucky I was at a centre that was close to Nairobi and I received colleagues that trained outside the city - outside the country, so they influenced me to think about other things out of medicine. Things go badly you can even venture into online jobs you know. They had their own connection there, so I can say they have really influenced me to survive out here. (Kenyan medical officer, KMO10 – L5 Public – Private MO)

The influence of ‘characteristics and hierarchy of career options and specialties’ (original category 10) on MOs’ career intention was supported by the Kenyan interview data, although less so by the Ugandan data. Some career choices and specialities appeared to be looked down upon or associated with stereotypical characteristics. For example, working in research was acceptable but most other options outside of clinical practice were not; ophthalmology was considered for women, neurosurgery and orthopaedics for men; psychiatry was looked down upon as a speciality choice. In the Kenyan setting, this not only led to certain specialities being less appealing to MOs as career choices but also to MO interns being less exposed to those specialities during their training due to internship coordinators’ neglect of those rotations.

… because we were the first lot that had the mandatory psychiatry rotation in internship, but then just from the onset, coordinator … (who) just told us like that’s not really going to happen. “I’m not going to give you time to rotate in psychiatry” … And then we’re also told that, you know, let’s like forget about the community health rotation which some of us had great interest in … It was sort of belittled like almost made fun of. (Kenyan medical officer, KMO30 – L4 Private not for profit – Business)

‘Workplace location, condition, resources and environment’ (original category 11) emerged as an important factor in the Kenyan and Ugandan data. This was in line with the original framework where this category was more commonly mentioned in LMIC settings. Three new findings from the interviews with MOs and consultants were as follows: (1) MOs preferred to work in the same facility after internship because familiarity made them more competitive when applying for jobs; (2) MOs would consider the specific strengths of their internship hospital (e.g. speciality) when applying for posts, with their intended career in mind; (3) workplace culture was also mentioned by Ugandan interns as influential.

One of the things they mention of course is, a pro-, a good working environment, a supportive working environment, an environment where there are available tools of trade. Some of them would mention that they feel limited staying in the government centres because of [a lack of] equipment and unavailability of supplies probably. (Kenyan consultant, KC09 – L6 Public - Paediatrics)

And so the hospital I worked at had a very good mental health department so I was also drawn to considering pursuing mental health so maybe psychiatry. Then they also were really good with research although you know … they don’t publish any research findings because of national security, so at least it also pushed me towards the path I’m in right now. (Kenyan medical officer, KMO29 – L5 Military – Research)

The aspect of ‘feeling valued by the organisation and the healthcare system’ (original category 12) was similar between the review findings and the Kenyan and Ugandan interview data.

It makes you think, why am I suffering like this in a place you are not appreciated, they only appreciate interns when they need you. My expectations before were to be a medical doctor and we honestly all need money but you realise that money isn’t there. You feel like you would do other things where you would earn and also get appreciated. (Uganda medical officer, UMO12 – Regional public – Unemployed)

One new category that emerged from both the Kenyan and Ugandan data was ‘witnessing and filling the health system gap’. Many MOs described witnessing challenges in the public health system during their internship. These included challenges with the national health insurance scheme, preventable deaths, delayed referrals, lack of resources, etc. that prompted interest in public health and non-governmental organisations as a career. In another case, the lack of orthopaedic surgeons in the national health system prompted an intention to specialise in an area allowing him/her to benefit the people and him/herself.

… there are so many patients that I ended up losing or … suffered complications simply because they didn’t have ability to access good care from the forefront. And it was like almost every day for me. I remember a case of a police officer who showed up with stage four cancer, and yet he had been to facilities all through that could have caught that cancer, but it just kept being missed … And for me I realised that yes, there are those who will play their role at this level 6 facilities, and there are those who need to play their role at the other level of healthcare cause, so for me, preventive medicine became important and crucial. (Kenyan medical officer, KM04- L5 Mission – Public MO)

### Theme 3: worry about the future

Kenyan and Ugandan MOs reported that they also considered the implications of their career choices on job security, training and professional development when making career decisions post-internship; consistent with ‘job market policies and changes’ (original category 13) and ‘future training and professional development opportunities’ (original category 14). MOs were concerned about job security, salaries and broad political context such as decentralisation, based on their experiences as interns. This influenced them to choose places where they could advance through training with consultant support rather than remain in MO positions for extended periods. While concerns over the length of training and salary for different specialities were common influencing factors in high-income countries summarised in the previous review framework, concerns over unemployment and underemployment were more pronounced in the Kenya and Uganda data due to the overall labour market context. For example, some Kenyan MOs chose particular specialities for greater chance of ongoing employment.

I guess it is also a mixture of personality also in as much as also it is a mixture of opportunities because there are some specialities that are easier to enter because they can take in a huge number of students and there are some that are a bit exclusive. (Kenyan medical officer, KMO21 – L4 Public – Private MO)

I chose [Hospital B] because of the similarity it shares with this place. First of all, it has all the four major disciplines, so that means I’ll be seeing inpatients, outpatients in any department of my choice. Two because for me… I didn’t want to just be there seeing OPD patients because then I feel like my medicine would rest and right now I kinda want to keep it active. (Ugandan medical officer, UMO10 – General Private not for profit – Private not for profit MO)

### Context and its impact on career intentions and decisions

This section describes the results from the inductive thematic analysis on how context shaped MOs’ career intentions and decisions. Participants reported a surplus of medical graduates from medical training schools, relative to public sector employment opportunities. Therefore, in both countries, we found a mismatch between MOs’ career intentions and actual career and employment decisions, as these were strongly influenced by job availability. Therefore, despite many interviewees intending to work in public hospitals, they often ended up employed in the private sector with poorer contractual terms and with a sense of resignation, as ‘it’s really just survival’ *(Ugandan medical officer, UMO16 – General Private not for profit – Private MO)*.

It’s not where I would want to be, there’s somewhere else I’d want to be. And if I were to choose, I would want to work in a government setting … an MO is private setting is also like exhausting … (Ugandan medical officer, UMO24 – National public – Private MO)

For me, I just wanted a job anywhere I could find, I didn’t have like, I was not able to have that choice, as in I was not spoilt for choice at that time, you just need a job where you can find one. (Kenyan medical officer, KMO02 – L5 Public – Public MO)

After you get your license, like you’re on your own. Now getting a placement after that, getting a job after that or where to work, it’s not up to you anymore. It’s whatever is available. That’s what you’re going to apply for, and then again, also after you get the job, you don’t get to pursue what you like most of the times, so you won’t really get to say, “I like I like orthopaedics, so I’ll go to an orthopaedic centre.” No. So whatever is available is what you will get. (Kenyan medical officer, KMO25 – L4 Public – Public MO)

Decentralisation of health workforce management in Kenya and local political landscape further added to the difficulty of securing a public sector job. Additionally, for the medical officers who entered the public sector, onwards deployment to subsequent jobs was often to a different location than they had hoped.

I applied and I got a job. But then you’re just posted, you’re just deployed. You don’t have a say that you want to work here, you want to work there. … I actually didn’t want to work in level 4 I thought I would do better in level 5 because I’m still young, and I wanted to still have my skills … So I wanted a place where there is a high flow of patients but unfortunately you don’t really get a say … You just deployed and there’s nothing you can do about it. (Kenyan medical officer, KMO23 – L4 Public – Public MO)

Lastly, some MOs who wanted to leave clinical practice and go into public health or management careers had to continue practicing as MOs while they pursued other forms of training such as Masters in Public Health for financial considerations. For those who wanted to start speciality training, significant changes in training policy and course fees made entry more difficult, with limited opportunities for government sponsorship.

I have always wanted to be a surgeon. So it has been clear in my mind what I intend to do after internship, but like I said, there are many challenges, things have changed. The structure of training has changed itself, talking about the universities it’s more expensive … , it’s much more difficult now to pursue these programs at postgraduate level. (Kenyan medical officer, KMO14 – L5 Public – Public MO)

## Discussion

We explored the complex set of factors influencing the career intentions and decisions of junior doctors in Kenya and Uganda, informed by the internship experience and career intentions framework we developed from a previous review [10]. We also considered how wider context impacted career choices. In the following paragraphs, we discuss our findings related to the conceptual framework, the specific influence of wider context on career choices, limitations of the study, and policy and practice implications.

Our empirical work in Kenya and Uganda largely supported the conceptual framework we developed previously from a review [10] – which did not include any papers from these two countries. All 14 categories from the original framework were relevant in the East African setting. This context is very different than in the primary studies included in the previous review (conducted mostly in high-income countries, such as the UK). We further extended the original internship experience and career intentions framework by adding a new category (witnessing and filling the health systems gap) that emerged from the data. Several categories such as ‘hands-on experience and real-life exposure’, ‘positive experience, confidence and readiness’, ‘relationship with supervisors’, and ‘workplace location, conditions, resources and environment’ were consistently mentioned and described as important by Kenyan and Ugandan participants when considering career intentions. However, the impact of Covid-19-related lockdowns and facility closures, and in Uganda, the specific 5–1 policy [19] seemed associated with interns reporting that they did not get enough experience, especially in some key specialities. Consequently, some sought to gain more experience through specific post-internship jobs, whilst others instead avoided these specialities. Interestingly, we did not identify further particularly pronounced effects of the pandemic on responses although there was perhaps more mention of interest in infectious diseases. Both ‘workplace location, conditions, resources and environment’ and ‘feeling valued by the organisation and healthcare systems’ were highlighted by interns as important in their career thinking. This was especially relevant to the public sector which is poorly financed, with facilities lacking essential resources, equipment and staffing [24]. In these settings, some interns felt like cheap labour, having to improvise and provide poor-quality care. Some decided to work in private or faith-based sectors or migrate to high-income countries where support and resources were more readily available to junior doctors. For some, witnessing national healthcare system challenges prompted them to go into research, public health and management. This specific category is new to the internship experience and career intentions framework, and we have added it as a new category ‘witnessing and filling the health system gap’.

In both countries, MOs’ career intentions did not necessarily match their final career and employment decisions as these were largely decided by job availability rather than personal preference. Many countries have expanded their health workforce training outputs in response to insufficient numbers of healthcare workers employed in the public sector [25]; however, our qualitative interview data support evidence that the increased investment in production may be limited by space to absorb these new graduates [26,27]. Many MOs reported that they hoped for a job in the public sector because of perceived better job security, but many ended up in private sector jobs with as locums or on short-term contracts. Recruitment in the public sector was further complicated by the local political landscape in some places, as reported in other literature [28–30]. In summary, we found that MOs’ preferences were largely determined by the job market. This may lead to disappointment and disillusionment in the medical workforce, exacerbating out-migration and may ultimately lead to a reduction in applications for medical training. It is therefore essential and urgent that countries accommodate greater public sector employment of junior doctors into workforce planning.

This study has several limitations. Firstly, most participants were working or hoping to work clinically at the time of the interview, therefore recruitment of those not working clinically or who had migrated might have captured additional findings. Secondly, our interviews were conducted as part of a larger study and before the original framework was developed, therefore we did not explicitly explore the framework categories in our interview guide. Consequently, we are less confident about the relevance of certain categories such as ‘workload, work-life balance, lifestyle’ and ‘well-being, emotional stress and the need to step off’ in Uganda, and ‘professional image and self-identity’ and ‘relationship with peers’ in both countries. These areas could be strengthened by additional data, but this was not possible due to time and resource constraints and could be the subject of future research. Lastly, our data indicated that internship and career choices are context-dependent, and therefore the findings should be interpreted against the broader context of government decentralistion (particularly in Kenya), as well as the mismatch between expanding numbers of medical school graduates and job availability. Future work could further test the evolving framework characterising internship influences on career choices, including our newly added category.

There are important policy and practice implications of these findings for internship training and workforce planning in Kenya and Uganda. This qualitative study supports evidence that there is an imbalance between supply and demand in the public sector, with relatively low absorption of medical officers into the public sector in these countries (less than 60%) [8,9]. Thus, despite many doctors expressing a preference to continue to provide public sector care, they are unable to. Preferred medical speciality and location are also limited by job opportunities. Similar challenges of unemployment and underemployment are seen in other African countries [26]. To make best use of training investments, countries should endeavour to ensure a more balanced supply and demand for MOs, with opportunities for doctors to pursue specialist training. To address interns’ concerns over job security and training opportunities, relevant job and training policies and opportunities should be clearly communicated during training. Lastly, countries need to review and evaluate their internship policies, especially rotation arrangements, so that interns receive sufficient exposure to different specialities with adequate supervision and resources. In Kenya special attention should be given to the integration of psychiatry and community health rotations, which according to our interviews were often overlooked by consultants and internship coordinators. In Uganda the Ministry of Health should evaluate the 5–1 policy to ensure that interns are well informed before choosing the major/minor speciality during their internship and that they gain essential competence for their minor as well as major specialities. Similarly, countries should try to ensure that internship could be carried out with sufficient exposure and adequate resources at all times, including during Covid-19, health worker strikes and other health systems disruptions.

## Conclusion

Drawing on interview data with Kenyan and Ugandan MOs and consultants, we explored how internship experiences influenced doctors’ career intentions. Our findings supported and expanded the internship experience and career intentions framework we developed from a previous review [10] – which did not include any papers from these two countries. However, the challenging medical workforce labour markets may dominate MOs’ career choices, as the final employment may be decided by job availability instead of personal preference. Such imbalance in supply and demand may lead to disappointment and disillusionment in the medical workforce and exacerbate workforce shortages, therefore it is essential and urgent that countries review workforce employment planning.

## Supplementary Material

Supplemental Material

## Data Availability

All data relevant to the study are included in the article or uploaded as supplementary information.

## Paper context

- Main findings: Internship experiences shape medical doctors’ career intentions in Kenya and Uganda, however job availability and context rather than preferences may dominate doctors’ career choices.
- Added knowledge: Career intentions differed from employment decisions due to specific contextual factors, most importantly a shortage of job opportunities in Kenya and Uganda
- Global health impact for policy and action: Countries need to improve workforce planning to ensure a better balance between supply and demand of labour so that doctors feel enabled to continue in their intended careers
