# Supplementary material for: The influence of internship training experience on Kenyan and Ugandan doctors’ career intentions and decisions: a qualitative study
Source: Glob Health Action. 2023 Nov 9;16(1):2272390. doi: 10.1080/16549716.2023.2272390 (PMC10653699; doi:10.1080/16549716.2023.2272390)
Supplement: Supplemental Material [file ZGHA_A_2272390_SM5400.docx]

**Supplementary appendix 1. Semi-structured interview guide**

**Questions (and probes) for medical officers:**

1. Please tell us about yourself (refer to the spreadsheet)
   1. What’s your current position? What type of occupation is it?
   2. Where did you complete your internship? Was it a public, private or mission hospital?
   3. Where did you undertake your undergraduate studies? Was it public or private?
2. How would you describe your internship experience?
   1. What are some of the things you have enjoyed most about your internship? Things least enjoyed? Can you give examples?
   2. Have you come across the term burnout, what does it mean to you? (state of emotional, physical and mental exhaustion caused by excessive and prolonged stress)
   3. How would you describe the level of support you received during internship? From whom?
   4. How do you think of patient safety in your internship hospital?
   5. How would you describe the level of preparation you got in medical school compared to the tasks you were given in your internship?
   6. How is it different from your intern friends who work in different hospitals?
   7. Did Covid impact your internship? health worker strike? government decentralization?
   8. What do you think can be done to improve your internship training and experience?
3. Can you tell me how did you choose to work in this current hospital/institution? What are the factors that influenced your decision to work here?
   1. Is this hospital/institution your first choice? Did you also apply to other jobs? Is it hard to find a job? How long did it take?
   2. Do you think there is a difference in terms of reputation of public/private/faith-based hospitals? How does that influence your decision?
   3. Did your preference for future career change during medical school and internship?
4. How did your internship experience influence your career decision?
   1. Do you prefer to work in certain hospitals or institutions because of your internship?
   2. How did your interaction with consultants/supervisors during the internship influenced your future plan?
   3. Did your previous relationship with your other colleagues during the internship hospital influenced your career decision?
   4. Are there any other social or political factors that influenced your career decision?
5. How safe do you feel about your current work environment, for you and for your patients?
   1. Your personal workload (any challenges accomplishing this?)
   2. In terms of the culture of safety in the workplace, and protocols if any (protective gear, waste disposal, essential work supplies and sundries, any changes with COVID-19)
   3. Adequacy of communication between colleagues for the care of patients (especially from senior to juniors, across professional boundaries e.g doctors to nurses)
   4. Teamwork (collaboration, cooperation, rather than competition)
   5. Participation in leadership decisions
   6. How and what would you like to see changed regarding safety at your workplace?

**Questions (and probes) for consultants:**

1. Please tell us about yourself
   1. What’s your current position? What specialty are you in?
   2. What hospital are you in? Is it a public, private or mission hospital?
   3. How long have you worked here?
   4. How many interns have you supervised? How many are you supervising now?
2. What do you think of the quality of the incoming interns? How well do you feel medical schools prepare interns for the job they have undertaken as an intern?
   1. What could be done to better prep students for internship?
   2. From your experience have you noticed any variations/differences in how well interns are prepared for their internship (i.e. Public/private universities or locally trained/those who trained abroad?)
   3. What should med schools do to better prepare students for their internship?
3. Do you think the interns are competent as a general medical officer after this one-year internship training?
   1. As a consultant/supervisor do you ensure interns have accomplished this?
   2. Do they have adequate opportunities to practice during the internship?
   3. How do you usually supervise and teach the interns?
   4. Do you receive any support from the hospital as a supervisor?
4. What do you hear about the experience of medical interns more generally?
   1. Some interns report high level of burnout and inadequate support received. Do you think that’s common? Can you give some example?
   2. How do you think the work conditions in this centre influence the learning experience of interns?
   3. What systems/structures are there to support interns during their internship? What types of support do you think should be provided to medical officer interns?
   4. Did Covid impact their internship? health worker strike? government decentralization?
   5. How was internship during your time? what is different now?
   6. What recommendations can you give to better prepare interns for their internship tasks?
5. How do you think interns decide on their future career after their internship? Why do you think interns choose to work in public/private/faith-based hospitals after they are registered and licensed?
   1. Why many interns opt for private practice?
   2. What do you think of the reputation of these hospitals?
   3. Is it hard for interns to find a job now? How long does it take to find a job?
   4. Are you involved in recruiting for this hospital? How does recruitment happen?
6. Can you broadly comment about safety at your current work environment, for you, your workers and for your patients?
   1. Your personal workload (any challenges accomplishing this?)
   2. In terms of the culture of safety in the workplace, and protocols if any (protective gear, waste disposal, essential work supplies and sundries, any changes with COVID-19)
   3. Adequacy of communication between colleagues for the care of patients (especially from senior to juniors, across professional boundaries e.g doctors to nurses)
   4. Teamwork (collaboration, cooperation, rather than competition)
   5. Participation in leadership decisions
   6. How and what would you like to see changed regarding safety at your workplace?

**Supplementary appendix 2. Comparing results from the meta-ethnography and Kenyan/Ugandan interviews with MOs and consultants**

| **Category** | **Meta-ethnography findings** | **Similarity and difference with Kenyan data** | **Kenyan quotes** | **Similarity and difference with Ugandan data** | **Uganda quotes** |
| --- | --- | --- | --- | --- | --- |
| **The “hand-on” experience and “real life” exposure** | Interns may or may not have an intention or interest during medical school and this could be either re-enforced or changed dramatically during internship. The “hand-on”, “real-life” exposure to clinical practice and different specialties make some realize they enjoy it, or in some other cases, they do not enjoy working with certain patients, there are unexpected responsibility with some specialties, or they don’t even want to continue practice medicine, as being a student and being a doctor has significantly different level of responsibility. The comparison between different rotations also helps interns decide which specialty fit them best. | Similarly, interns’ interests could be cemented during internship when they enjoy certain rotations or sometimes made them realize “not for me”. CMEs was also mentioned as one source of exposure. | *M24 (L5 – Public – Public MO)*  After when I started my internship, I started with int Med yeah. And of course I was excited about it 'cause I was like, I could actually practice how I could become a physician in future. But after I think, like I said, week 6-7 I actually got bored because I realized unless something new happens, it's the same thing every day, over and over again and I just lost interest after a while. Then I went to pediatrics after that. Again, after a while I got bored because, I don't know. It was also the same same thing every day. Then I went to obstetrics and I never actually thought I would enjoy obstetrics. But I did. And that’s actually what I’m going to practice in future. So, that's where that's where I am at right now. I am passionate about it. I enjoy it every day. I actually don't mind doing it. And also in line with my other passions. You know family planning, contraception use, early pregnancies, so it just it. I don't know, it just worked out in that in that direction. I think I don't want to comment about surgery because I don't think I had a good feel of surgery. Like I said, because of COVID. | Similarly, interns’ interests could be cemented during internship when they enjoy certain rotations or vice versa. | *UMO21 (General Public – Private MO)*  Because internship puts you in the actual workplace. You know, when you're in medical school, you are seeing things from outside almost. You're studying, and you're on seeing the wards, but you're not really involved. So when you go into internship, you’re like in the real life medical situation, you have decisions to make, you’re the one in charge of particular tasks and stuff like that. So it makes you see how you could potentially be like living for the rest of your life. |
| **Positive experience, confidence and readiness** | After successfully managing patients in different rotations, interns may feel confident and ready to further develop skills in certain specialty and sometimes with supportive consultants and team they rebuild previously damaged confidence; or on the contrary, negative experiences with lack of support could lead to inters feeling uncomfortable, losing confidence and not ready to further continue practicing or enter specialized training. | Similarly, good experiences in certain rotations draw interns’ interest towards that specialty while bad experiences turn people off.  In addition to the review findings, in some cases interns may not get enough experience or exposure in certain areas thus want to fill those gaps and get more exposure post-internship | *C05 (L5 – Public – Surgery)*  And also some of the things which they also tend to, to move which they which they used to choose their direction, is their strengths. There are some who are very, very good in theater. So you find that when a county announces for employment and they say that these young guys will go to maternity, some will withdraw and say they were not good in doing CS and they are not comfortable in doing CS, they don't want to be CSs and they will drop, the ones who are comfortable will actually go for that employment. So I think also strengths and weaknesses tend to influence how they will go.  *M02 (L5 – Public – Public MO)*  I had wanted to major in surgery because I feel like it really not exhausted my potential during my internship at the surgery department, so I wanted to start with surgery so that I can learn as much as I can but that was not possible then. | Where interns did not get adequate experiences or exposures in certain areas due to institutional arrangement (Uganda’s 5-1 policy), interns are not confident in those areas and would turn away. | *UMO12 (Regional Public – Unemployed)*  Actually right now I can’t do anything in surgery, I am not confident enough. It is more of biasing for example you go for something you are not passionate about for five months and then get just a month for something you are passionate about. I was at the surgical ward but with no theatre during COVID. we just did medical management of surgical patients. |
| **The workload, work-life balance, lifestyle** | Interns prefer more manageable workload and healthier work-life balance. During internship, they may experience or observe the actual workload/lifestyle of different specialties, which the actual situation might be different than what they expect (e.g. GPs also work stressful and long hours). Interns will choose careers (specialties, country of work) that fit their preferred lifestyles (some can also sacrifice) and whether it will be compatible with their future personal life of e.g. having a family and children, which also has a gender aspect to it. | Similarly, interns prefer more manageable workload and work-life balance which reflect in different specialty choices – from their own experiences or observations of their senior colleagues.  However, there is also a difference in workload between public and private hospitals which influence people’s choices. | *M13 (L5 - Public – Public MO)*  I think I had a feeling that practice was not for me during med school, but I, of course when you're in school, you really don't have a very clear idea of what the outside world is and what your opinions of this were.…But when I came for internship is when I confirmed that for sure this wasn’t for me, uh... So it would not be totally true for me to say that it is internship that changed my mind, but it really confirmed that it wasn’t for me, especially the workload at a public hospital that is so big. You, it really confirmed it and it made me certain I absolutely do not want it.  *M16 (L5 – Public – Public MO)*  I wanted a public hospital because one when you compare the workload in private to the one in public, private is, okay from my experience, private it has a lot of, it has a heavy workload. So public is always my better option. | Interns prefer specialties and also hospitals that have less workload due to their experiences during internship. | *UMO02 (General PNFP – PNFP MO)*  As an MO, you have a duty which can be maybe up to 5:00 o’clock. But as an intern, you stay for a longer tome waiting for a hand over. For me I would prefer government because I know I will have time for myself. If in a week I have 3 duties, which is even more because sometimes they have less than those duties. I would also want that rather than working in a PNFP where by you will get 2 days off or even 1 day off which is very different. |
| **Well-being, emotional stress and the need to step off** | Internship training is stressful, exhaustive, and sometimes also lonely, especially in certain rotations. Interns feel like they are on a “conveyor belt” or “treadmill” all the time, and will prefer careers that they can bear, and in some situations “step off” and take time out of training to alleviate their stress and exhaustion. | Similar with the review findings, interns would choose to “take a break” after internship because of their internship experience was really stressful (M30 who chose to work in business).  One additional finding is that some specialties were also described as very stressful and “living on the edge” which turned people off. | *M08 (L4 – Private – Private MO)*  For right now I know based on how my obstetrics rotation went. I mean, it was great and everything…great mentorship, I learned a lot but I don't think I'm really cut out for that life. You know, living on the edge being alert on call at any point, I know it's not, it's not the life I want. I mean, I want to be able to, I know this is the weekend, patients are stable. Yeah, I can sit back and relax. Nobody's going to have, you know, premature rupture of membranes at 3am in the night. You know, things like that. Like different internship I know now, that's not my kind of environment. So that one is out for me. I know and I okay, now based on internship and maybe what I'm doing currently. I normally empathetic, I used to love oncology I think am cut out for that line of work. Yeah, it really shaped me… | Similarly, interns prefer to choose careers that are less stressful.  However, this category is not strongly supported by data. | *UMO17 (General PNFP - Unemployed)*  Because of the way internship is set up, you get burnt out a lot and then you just become - when you start applying for jobs, you become really sceptical about working too many hours because you don't want to ever be that burnt out again. |
| **Image of professions and self-identity** | There are public and social status, respect and characteristics linked with certain specialties that are being reminded to doctors and also during internship. Interns may find this time to reflect and think whether their proposed career fit their self-identity, whether choosing certain specialty will make them being considered as “not a proper doctor”. This is also linked with the hierarchy of career options and specialties. | While professional image and self-identify are similar between the review findings and the Kenya data, what’s new from the Kenya data is some specialties were considered for other lower cadres thus “not for doctors”.  However, this is the weakest category and not supported by adequate data. | *M20 (L4 – Public – Mission MO)*  Well just a general feeling, not really from my supervisors or anything. But I don’t know, guys don’t like doing anaesthesia. Yeah, they feel that there is no work for them, they only work in ICUs, you know there was a course that was introduced, it is for clinical officers. So they specialize in anaesthesia and then nursing, nurses can also do, become an anaesthesia nurses. So these days, most of the work is done by the clinicians. And for you to get employed as an anaesthesiologist it is not that easy and most of the other medical officers they say, quote and quote the feeling is the, they say lazy or something people don’t like to read. | Again what’s new from the Uganda data is some specialties were considered for general doctors thus not worth pursuing.  However, this is the weakest category and not supported by adequate data. | *UMO23 (General PNFP - Unemployed)*  Then also the whole time I was in the outpatient seeing children maybe since I’m just an intern, but everyone I saw was just cough, flu, malaria, fever. So I feel like so many people would treat that. So if I did that Paediatrics I wanted to do, yes the patients would be there but what you’re treating is what any general doctor would treat so that’s why I feel like I should do something special like ENT, that if this patient has this and this, only an ENT surgeon can do that work so people will look for you other than doing something that everyone can do. |
| **Relationship with supervisors/consultants** | Consultants and supervisors are very influential in interns’ career choices. Enthusiastic and supportive supervisors could be mentors, role models, giving career advice (even lobbying) and interns will “want to be like him/her”. In some other cases, there could be insufficient or no role models, or poor ones that bully interns, do not adequately support interns of their practice and career choices, or performing poor clinical practices and interns will “not want to become such doctor”. | Similarly, this category is considered the most important one with lots of data. Interns receive encouragement, mentorship, career advice from their consultants and “want to be like them”, or witness “bad” examples (e.g. no professionalism) or bullying thus changed their career intentions. It should be noted not all respondents say it made a difference.  Two additional findings from the Kenya data are: (1) Interns may witness “successful” consultants and “struggling” ones which will affect their intentions. While this come up in the review for “relationship with senior colleagues” it wasn’t mentioned much from supervisors.  (2) Interns who perform well could directly secure locumming opportunities from their supervisors. | *M08 (L4 – Private – Private MO)*  But then when I transfer transition to internal medicine, it was it was a hell. I was first of all, racially profiled. Yes, I was racially profiled and at some point, it almost generated to physical abuse. I had a file flung at my face. That’s story for another day.  So what end up happening, in as much as I loved internal medicine, I knew I deep down wanted to be a physician. Those four months actually five ‘cause I did the three months and then they added me an extra two months maybe for working for people for free. But those last four months were torture. I left [Hospital N] knowing I will never, ever, ever touch internal medicine again, because I didn't want to be liked to those two bullies.  *M11 (L4 – Public – Public MO)*  My interaction with my consultants and my, really...it did because one, I will- First, of all, let me just say that money is normally a very powerful incentive for anything. So some of my lecturers, my undergraduate years seemed to be living very comfortable lives for example…Another example of a successful radiologist is another one who I also interacted with very closely. He was not my lecturer, but we interacted with him in the ward, extracurricular life. He is called Dr. [Name]. He's also very, you know, financially comfortable. So that is one of the, that was one of the, that one to be honest, also inspired a lot of interest on my side. | This category is again considered the most important category and supported by most data. Mentorship and support from consultants were constantly mentioned.  Similar with the Kenyan data, (1) interns can interact and see other consultants being successful or broken; and (2) get linked up with jobs from consultants either in private sectors or locumming. | *UM08 (General Public – Unemployed)*  Well you’d interact with someone and see the stress of their practice on their faces right or in their words then you know like I can’t do this. Then you see someone who is enjoying it and you’re like, you know, I want to be this happy. |
| **Relationship with peers** | Peers are important social relationship during internship training that can support each other. Interns make career decisions based on the information sometimes generated from peer networks, and interns may want to make choices that are “validated” by their peers and follow others’ recommendation or what everyone else are doing. Interns may also want to continue working in certain locations or specialties because they want to maintain established personal relationship. However, there are also competition between peers, such competition and “elbowing one’s way into the theatre” may deter interns’ interest into certain career, and some may decide to leave so that there could be less competition for their peers. | While the Kenya data also suggested that interns talk to peers to understand and share working experiences in different hospitals and potential job opportunities, we did not identify any data relating to “validation” by peers, maintaining “personal relationship” or competition with peers. | *M10 (L5 – Public – Private MO)*  By the way we lack as Kenyan, I can say as a Kenyan intern at that time, without colleagues coming from outside, who had studied abroad, we were very few and only goes to centers that are very close to the city, you may lack to get experience of outside Kenya. So I can say I was lucky I was at a center that was close to Nairobi and I received colleagues that I trained outside the city- outside the country, so they influenced me to think about other things out of medicine. Things go badly you can even venture into online jobs you know. They had their own connection there, so I can say they have really influenced me to survive out here. They haven’t influenced me to what major on what I will do out there, but surviving without an employment. | Similarly, we also did not identify any data relating to “validation” by peers, maintaining “personal relationship” or competition with peers. | *UMO17 (General PNFP – Unemployed)*  I think for me, you know, how I've been telling you that I realized I don't like seeing patients every day. It was interesting seeing people who actually like enjoy like the day-to-day like the drudgery of every day, and actually like finding purpose and enjoying it. It was interesting. It made me realize like I can actually do something I like. I don't have to do something I don’t like just because it is what’s on the table. |
| **Relationship with senior colleagues and the team** | Other senior colleagues and the healthcare team are also key to career decision-making. Interns prefer teams that are supportive, friendly, approachable, feeling welcomed and valued, and demonstrate similar characteristics – and reject ones that are unsupportive. Interns also could seek advice from other colleagues like specialties trainee to get more familiar with working practices and training requirement, or sometimes just “witnessing registrar that are broken” and therefore shift career plans. | Similar with the review findings, interns draw experience from senior colleagues who may be immediate past interns and seek job opportunities. Interns also mentioned nurses being inspiring for their career intention.  Registrars were not mentioned in our data which might be due to different training systems in Kenya (registrars tend to work in a very few L6 hospitals). | *M12 (L4 – Public – Private MO)*  Let me say this, I might be biased, but the best time I ever had was, within maternity ward had nurses who were there who were very hard working and they taught me a lot of things even as an intern, as a medical officer and I can never take that away from them. Of course, you know, if you work in such an environment, you'll also find nurses who would prefer for you to rush a patient towards CS, rather than you know follow up the patient and check them up, but with maternity, they were very hardworking nurses, very, like, they are ready to teach. But in pediatrics, the nurses were the same, ready to help, ready to teach, so, that's also like jolted me to wanting to pursue a career in pediatrics. | Similar with review findings, and registrars were mentioned by interns as they witness their experiences. | *UMO24 (National Public – Private MO)*  Now obstetrics, if I was to ever want to do obstetrics, I would never want to do it from [Hospital A] because postgrads are equally burnt out as the interns…. And it actually influences the choices people make. |
| **Relationship with patient and the community** | Through working with patients and the community, interns may realize if they like such interaction, interns may also feel like they are integrated to the community and want to continue serving the community that decided their career choices. | Similar with the review findings, interns may decide on their career intention based on experiences interacting with patients or witnessing the needs of the patients. | *M12 (L4 – Public – Private MO)*  Okay, so for me, I had a choice between internal medicine, obstetrics, gynecology and pediatrics. What I realized for me, I'm driven more into to pediatrics because parents listen, parents really take care of their children, the staff at the pediatric ward were very responsive to any complaints rather than the dismisses complaints in the medical wards or in the surgical wards. | Ugandan data mostly suggested that interns may realise that they did not enjoy patient interaction thus prefer a career that does not involve direct patient care. | *UMO17 (General PNFP – Unemployed)*  I think like that more you do the work you realise that maybe I should just go into epidemiology and leave medicine alone. Because medicine is quite exhausting, like the practice of seeing patients every day, every day, every day. Yeah, it just reaches a point and it is so unappealing. So unappealing. I've actually even been considering a career in epidemiology and I just leave patients alone completely.  *UMO23 (General PNFP – Unemployed)*  And then also the research, the less clinical work, I thought of that because so many times I would wake up and not want to see patients but you would have no option, patients are there and you have to see them. And I feel like I would be happier doing my own work, very little contact time with patients.So that’s why I keep thinking I should be going away from clinical practice. |
| **Characteristics and hierarchy of career options and specialties** | There are said and unsaid characteristics and hierarchy of different career choices and specialties. Interns constantly hear consultants, senior colleagues or their peers commenting or bashing on some specialties like “just a GP” or “not worthwhile” or some specialties being more gendered e.g. surgery being a male-dominated specialty and females are discouraged from it. Interns sometimes even say this among themselves or have to hide their preferred intention for future career. This inevitably influenced interns’ career decisions. However, not everyone is affected by this (ref 167). | Similarly, certain career choices and specialty would be looked down upon or associated with certain characteristic: for example working in research is acceptable but all other options outside of clinical practices are not; ophthalmology is considered for women, neurosurgery and orthopaedics for men; psychiatry being looked down upon. What’s new is that this not only leads to certain specialties being less appealing, but also leads to lead interns are less exposed to different specialties from the start. | *M30 (L4 – Mission – Business)*  because we were the first lot that had the mandatory psychiatry rotation in internship, but then just from the onset, coordinator, who's a surgeon just told us like that's not really going to happen. “I'm not going to give you time to rotate in psychiatry” … For us we were told like we'll only do Friday psychiatry clinics, outpatient, with the family medicine consultant, and that was final, no discussion! And then we're also told that, you know, let's like forget about the community health rotation which some of us had great interest in and, you know, it was just like since they're not really entirely hands on, entirely clinical, entirely hospital based, areas of interest, then it's not important, you know, you're supposed to be a clinician… So we actually had one of us that was really interested in psychiatry and she was really, she was really sad about it, because every time we raised the issue, you know, she was just shut down continuously every time she tried to talk about it and say, you know, some of us have an interest in psychiatry. It was sort of belittled like almost made fun of… | This category was not supported by adequate data, and only one mentioned certain specialty having characteristics that influenced his/her decision. | *UMO22 (Regional Public – Unemployed)*  May be social factors, like the way society views. For example in paed, people would prefer female pediatricians. So it could a minor factor. |
| **Workplace location, condition, resources and environment** | Interns are also drawn towards workplaces that have good facilities and resources, supportive environment, and high morale. | This came up quite prominently in the Kenya data. Similar with the review findings, interns are drawn towards well-managed facilities with adequate resources and less bureaucracy.  What’s new are: (1) Interns prefer to work in the same facility after internship because they are familiar with the setting which also make them more competitive when applying for jobs; (2) Interns will consider what the internship hospitals are good at (e.g. which specialty) and use that to cultivate their career interest. | *M11 (L4 – Public – Public MO)*  Going into internship, I had an ideal place where I would have loved to work. But after internship, I realized that my internship centre was not that ideal place. So I perhaps, I would have wished to work at a private facility, a facility such as [Hospital L], where I would have the support of all these all be working departments and functional units. But I think the biggest impact that internship had on me was that I would have wished to...to make where I did my internship a better place. I would have wished to inject my energies.  *M13 (L5 – Public – Public MO)*  Number two, when you worked or done internship in a county, you want to go, when it comes to hiring, you have an advantage because now in the panel you can just explain to them that I was here, I have worked here, I know the doctors and I want to remain here and serve the community. And easier sell compared to someone who was because they feel that you were trained by their doctor or hospital, so you tend to do better.  *M29 (L5 – Military – Research)*  And so the hospital I worked at had a very good mental health department so I was also drawn to considering pursuing mental health so maybe psychiatry. Then they also were really good with research although you know with the military they don't publish any research findings because of national security, so at least it also pushed me towards the path I'm in right now. | This also came up prominently in the Uganda data where interns draw towards facilities with adequate resources.  Additionally, workplace culture was also mentioned by interns that influence their decision. | *UMO17 (General PNFP – Unemployed)*  With faith-based hospitals there's also a relative amount of job security but having worked in one, I think there's a lot of hypocrisy. Can I say? There’s this idea that they're here to save lives and they're cheaper than other private hospitals. There’s this idea that they are better than private hospitals yet they really are not. There are here to make profits, just as much as the private hospitals. So people come expecting a subsidized rate and then they end up paying so much without the upfront expectation of spending a lot of money. Yeah, so that hypocrisy rubs me the wrong way. So right now, I would rather work in a private hospital and a government hospital. That's really what I would like to do. |
| **Feeling valued by the organization and healthcare system** | Interns sometimes feeling undervalued and under-appreciated by the organization and the healthcare system. They felt they are considered as “cheap labour” for service provision. They are sometimes frustrated by the administration that lack accountability, does not care about their wellbeing, and consider interns as hassle if they raise concern. Therefore interns may consider choosing other work organizations (private sector, another country) for their future career. | Similar with the review findings, interns prefer to choose employers (sector/county) where they are supported and being valued. | *M11 (L4 – Public – Public MO)*  Because one of the things that have been, especially in my county, I have seen it. I saw it before internship, during internship and even now that I am working there as an MO, serious salary delays, the serious salary delays are so bad but they have the effect of making it seem as though you know you miss...You miss salary for certain months. So, one of the considerations was, and it has been a very prominent consideration. We discussed it is not only among ourselves, but in the wider doctor community people who advocate for immigration for other countries to pursue your studies and your dream careers there. Or at the least working in the private sector. | Similarly, interns considered to find other career opportunities where they feel would be more appreciated. | *UMO12 (Regional Public – Unemployed)*  It influenced it negatively. It makes you think, why am I suffering like this in a place you are not appreciated, they only appreciate interns when they need you. My expectations before were to be a medical doctor and we honestly all need money but you realise that money isn’t there. You feel like you would do other things where you would earn and also get appreciated. |
| **NEW: Witnessing and filling health system gaps** |  | This is a new area. Many MOs described witnessing the systematic challenges in the public health system during their internship including the NHIF, preventable mortality, referral, etc. thus wanting to pursue public health as a career. For another MO, s/he witnessed that the county is lacking in orthopaedic surgeon thus having an intention to specialize in that, which allowed him/her to benefit the people and him/herself. | *M04 (L5 – Mission – Public MO)*  Just the patients and just what they have to go through, you know, like the way Kenya is set up, before a patient shows up at like [Hospital J] they have gone through several other cadres or hospitals lower. And there are so many patients that I ended up losing or just having to, that suffered complications simply because they didn't have ability to access good care from the forefront. And it was like almost every day for me. I remember a case of a police officer who showed up with stage four cancer, and yet he had been to facilities all through that could have caught that cancer, but it just kept being missed, and by the time he showed up, he was in stage four and he was just 35 years old. And that really bothered me because, one, he had ability to access that facility, they had the ability to access healthcare. So he accessed it, but wherever he accessed it, the facilities were not well equipped to make timely diagnosis. And there were so many of such stories in different forms. And for me I realised that yes, there are those who will play their role at this level 6 facilities, and there are those who need to play their role at the other level of healthcare cause, so for me, preventive medicine became important and crucial yeah, I think it's just the experiences I encountered. | Similarly, Uganda MOs described witnessing systematic challenges and hoping to work on careers related to health system changes. | *UMO10 (General PNFP – PNFP MO)*  So I’m thinking our health systems - we are trying, but we need some health system strengthening. I feel like we need better… you’ll find that many facilities, a number of facilities don’t have the necessary things to manage patients so they keep referring them to higher facilities, yet those facilities would have been able to actually do it, but they can’t because they lack the necessary support. So we do need health system strengthening, health system support. So actually that may be one of the things that is influencing my career decision. |
| **Job market polices and changes, job security: will I get a job** | The job market polices for example changes to junior doctor contract, government cost-cutting are felt even closer during internship period, this will lead to interns choosing specialties that are more employable in the future or workplaces that have more certainty. However, it’s not always a strong influencer and sometimes it’s hard to predict. | While junior doctor contract was not specifically mentioned, interns are concerned with decentralization, job security and which specialty seems more employable. | *M08 (L4 – Private – Private MO)*  So even right now you're thinking, okay for example maybe I love, let’s say I love psychiatry, but you're looking at it in terms of even after I struggle through will I have an income after? Am I employable? So it's, it's what has really shaped the decision. I know of so many people who are like really passionate, for example, about being physicians, but somebody tells you, ‘okay, so become a physician and get out and then?’ So they’ve wound up in things like obstetrics, they’ve wound up in things like EMT. Yeah, just ‘cause of that, you know, thinking in the next five years, will I be employable? Will I have somewhere to have my footing? I have, I have a friend who finished surgery, and has been job hunting for the last six months. You go somewhere somebody tells you, we can't employ you because we can't afford to pay you and we can can’t employ you as a medical officer, I mean, you're overqualified. | Similarly, interns were concerned with job security and salary. | *UMO05 (Regional Public – Public MO)*  Of course, we have the experience, we have the passion of caring for patients to treat and save lives but again we need to survive. So as a doctor, I cannot be in a place where a boda boda man earns more than me.  *UMP17 (General PNFP – Unemployed)*  I think, I think everyone knows that working in Uganda as a doctor is quite difficult. That thing where you like you do two jobs just to make ends meet, where you just - even with the two jobs you’re just barely touching the surface and you considering should I do a third job, should I do a masters, should I maybe change a career? … Yeah, it really does make you think about like switching to a career where there is - because I really don't want to work two jobs. |
| **Future training and professional development opportunities: will I get advanced** | Interns will choose career options that will allow their future training and professional development, based on what they experience with the healthcare team and what they hear about in other settings. Interns won’t choose pathways that limit personal development, or jobs that are low-quality and “no one else want”. | Similarly, interns will choose places where they could get further advancement through training and working with consultants, where they are not “chronic MOs”. As county governments are no longer sponsoring MMed training, public sector seems less appealing as an option. Additionally, interns are more pragmatic and are leaning towards specialties that are easier to get into based on their experiences | *M16 (L5 – Public – Public MO)*  We don’t know what will happen after the three years because there are some who after the three years they will get confirmation of permanent jobs and there are those who don’t, so it depends with the county that you are in because there are some counties who convert from three year contract to PNP but basing on the fact that most of them are yet to have employees at their facility for more than three years, we do not know what will happen but it is pushing people away from doing, from been a chronic MO let me say a chronic MO. | Similarly, interns will choose place where they will get further training and advancement. | *UMO10 (General PNFP – PNFP MO)*  I chose [Hospital B] because of the similarity it shares with this place. First of all, it has all the four major disciplines, so that means I’ll be seeing inpatients, outpatients in any department of my choice. Two because for me... I didn’t want to just be there seeing OPD patients because then I feel like my medicine would rest and right now I kinda want to keep it active. |
